# Methanol Production by a Broad Phylogenetic Array of Marine Phytoplankton

**DOI:** 10.1371/journal.pone.0150820

**Published:** 2016-03-10

**Authors:** Tracy J. Mincer, Athena C. Aicher

**Affiliations:** Woods Hole Oceanographic Institution, Department of Marine Chemistry and Geochemistry, Woods Hole, Massachusetts, United States of America; University of Copenhagen, DENMARK

## Abstract

Methanol is a major volatile organic compound on Earth and serves as an important carbon and energy substrate for abundant methylotrophic microbes. Previous geochemical surveys coupled with predictive models suggest that the marine contributions are exceedingly large, rivaling terrestrial sources. Although well studied in terrestrial ecosystems, methanol sources are poorly understood in the marine environment and warrant further investigation. To this end, we adapted a Purge and Trap Gas Chromatography/Mass Spectrometry (P&T-GC/MS) method which allowed reliable measurements of methanol in seawater and marine phytoplankton cultures with a method detection limit of 120 nanomolar. All phytoplankton tested (cyanobacteria: *Synechococcus* spp. 8102 and 8103, *Trichodesmium erythraeum*, and *Prochlorococcus marinus*), and Eukarya (heterokont diatom: *Phaeodactylum tricornutum*, coccolithophore: *Emiliania huxleyi*, cryptophyte: *Rhodomonas salina*, and non-diatom heterokont: *Nannochloropsis oculata*) produced methanol, ranging from 0.8–13.7 micromolar in culture and methanol per total cellular carbon were measured in the ranges of 0.09–0.3%. Phytoplankton culture time-course measurements displayed a punctuated production pattern with maxima near early stationary phase. Stabile isotope labeled bicarbonate incorporation experiments confirmed that methanol was produced from phytoplankton biomass. Overall, our findings suggest that phytoplankton are a major source of methanol in the upper water column of the world’s oceans.

## Introduction

Oxygenated volatile organic compounds (OVOCs) are involved in key atmospheric processes on Earth. As one of the most abundant non-methane hydrocarbons in the lower atmosphere (troposphere) [1], methanol serves as a carbon and energy substrate for a specific class of microbes (termed methylotrophs) possessing the ability to use heteroatom single carbon compounds (C1 compounds) as a sole carbon and energy source [2]. Methanol also plays a significant role as the dominant OVOC in the atmosphere where it is known to produce ozone in the troposphere [1, 3]. Methanol sources are well-known in terrestrial ecosystems where about 80% of atmospheric methanol production is attributed to plants, and to a lesser degree decaying plant material and anthropogenic activities (1).

In the ocean, picoplankton driven methanol turnover times can be rapid, ranging from an estimated 7 days in northeast Atlantic coastal waters [4], to as low as 1 day in oligotrophic tropical northeast Atlantic samples [5]. Additionally, major groups of bacteria have been discovered to be obligate methylotrophs, such as the abundant members of the beta-proteobacteria, OM43 [6]. Altogether, these findings highlight the importance of methylotrophy in the marine microbial food web. Although methanol serves as an important microbial substrate and climatologically relevant compound, sources of methanol in the marine environment remain unknown.

The predominant source of methanol on our planet is currently understood to be a waste product of terrestrial flowering plants which are also known to harbor abundant populations of methylotrophic bacteria associated with their root systems and particularly on the underside of their leaves where methanol is the primary C1 compound excreted from the enzymatic hydrolysis of pectin [7]. Investigation of methanol sources has focused almost solely on terrestrial systems with about 50–280 teragrams (Tg) per year contribution to the atmosphere from primary biogenic sources. It has been posited that the world’s oceans could be a gross source of atmospheric methanol (up to 30 Tg per year) [3] and a recent study suggests that the Atlantic Ocean alone could contribute at least 3 Tg per year [8]. Moreover, computer modeling efforts estimate ocean sources of methanol to be at least equal to the atmospheric input of methanol by terrestrial plants, particularly over oligotrophic, open ocean regions [9]. Ocean sources of atmospheric methanol have recently been put into question, however, where a trans-latitude Atlantic study reported all open ocean stations to be atmospheric sinks for methanol [10].

Regardless, methanol production in the oceans is estimated to be large to fuel dependent methylotrophic microbial biomass and account for gaps in the atmospheric budget [3, 10]. For example, the need for a missing oceanic source of methanol to offset a mass balance of two orders of magnitude, if atmospheric deposition was considered the only other source of methanol, was recently highlighted by the same trans-latitude Atlantic report [10]. Only one study to date has been published implicating phytoplankton as a methanol source where various phytoplankton cultures were surveyed for methanol production ranging from a coccolithophore, two dinoflagellates, a haptophyte and a cyanobacterium; although an important step forward, this study was performed on presumably xenic phytoplankton and was largely qualitative, leaving the actual sources of methanol open to speculation; however, production of micromolar amounts of methanol were estimated from phytoplankton cultures in that study (originally reported in [11] and later reprinted in [3]).

Given that phytoplankton in the world’s oceans account for nearly half of the primary productivity on our planet [12] and the importance of methanol in the atmosphere, and as a carbon and energy substrate for microbial methylotrophs we sought to better characterize this potentially important source. Specifically, we show that a wide range of phytoplankton produce significant amounts of methanol, and that methanol uptake was undetectable by these axenic strains. Through stable isotope incorporation experiments and comparative elemental analyses we further show that methanol: 1) accounts for a significant portion of phytoplankton cellular carbon, and 2) is produced *de novo* from algal biomass, suggesting that phytoplankton are a major source of methanol in the marine euphotic zone.

## Materials and Methods

### Microbial strains used in this study and growth conditions

All phytoplankton strains were maintained and grown on a 14:10 light:dark cycle regime in temperature controlled lightbox incubators (Percival Scientific, Boone, IA, USA) using cool white fluorescent bulbs and a photon flux in the range of 25–100 microeinsteins per second per square meter (μE m^−2^ s^−1^) specified for each cultivar. *Rhodomonas salina* strain CCMP1319 (grown at 18°C, 60–75 μE m^−2^ s^−1^), *Nannochloropsis oculata* strain CCMP525 (grown at 15°C; 25–35 μE m^−2^ s^−1^), *Phaeodactylum tricornutum* strain CCMP632 (grown at 18°C; 25–30 μE m^−2^ s^−1^), *Emiliania huxleyi* strain CCMP371 (grown at 18°C; 25–35 μE m^−2^ s^−1^) were all obtained from the National Center for Marine Algae and Microbiota (NCMA, East Boothbay, ME, USA) as axenic cultures and maintained in f/2 –Si medium. The cyanobacteria *Trichodesmium erythraeum* strain IMS101 (grown at 27°C; 50–55 μE m^−2^ s^−1^) and *Synechococcus* spp. 8102 and 8103 (grown at 23°C; 80–100 μE m^−2^ s^−1^) were obtained from the WHOI Culture Collection and maintained in RMP or SN media, respectively [13]. *Prochlorococcus marinus* strain MED4ax (23°C; 35–45 μE m^−2^ s^−1^) was a kind gift from the Chisholm laboratory at the Massachusetts Institute of Technology and maintained as recommended [14] in PRO99 medium purchased from the NCMA. The methylotrophic bacterium *Methylophaga marina* ATCC 35842 was purchased from the American Type Culture Collection (Manassas, VA, USA) and maintained at room temperature in f/2-Si medium amended with 0.2 μm filtered methanol, 0.3% final concentration, 0.2% agar was added for solid medium as needed.

### Measurement of Methanol

A purge and trap gas chromatography mass spectrometry method (P&T-GC/MS) was adapted to measure methanol as previously described [15], with modifications. The P&T-GC/MS system used in this study was an Agilent 6850 GC with a single quadrupole 5975C MSD equipped with electron impact ionization, coupled to a Tekmar Solatek 72 vial autosampler with liquid and solid sample processing capabilities, and a Tekmar Stratum sample concentrator (Agilent Technologies, Santa Clara, CA, USA) fitted with a K-type Vocarb 3000 trap (Sigma-Aldrich, St. Louis MO, USA) described below. Parameters for the Tekmar Stratum sample processing are detailed in the S1 File. Various types of traps were tested and the K-type Vocarb 3000 proprietary resin (Supelco, Bellefonte, PA, USA) which is specifically designed to sorb low molecular weight alcohols and ketones from water was determined to be optimal for our methanol analyses. Chromatography was optimized using a ZB-Wax capillary column (60 meter, 0.25 mm ID, 0.5 μm film thickness; Phenomenex, Torrance, CA, USA) in combination with a 1:5 split ratio allowing for the high expansion of methanol in the injector chamber yielding highly reproducible retention times (within 0.1 minutes) and low error in sample technical replicates, typically less than 5%. Detection was performed in SIM-Scan Mode where the total ion current (TIC) in the range of 20–500 m/z was monitored together with Selective Ion Monitoring (SIM) at 31 m/z (the major fragmentation ion for small alcohols and ketones, including methanol) and at 33 m/z (the specific molecular ion for ^13^C-labeled methanol). Standard electron impact-MS ionization parameters of 70eV were used. Identity of methanol signal from samples was verified by comparison of retention time and fragmentation pattern to an authentic standard. The methanol fragmentation patterns of all samples were also matched to the NIST GC/MS library (version 2008) for additional confidence. Chromatography conditions were optimized for methanol: 40°C temperature hold for 14 minutes followed by a temperature increase of 150°C per minute to a final temperature of 240°C maintained for 4 minutes to clear sample residue off the column; helium flow was maintained at a constant flow of 0.8 mL per minute. Standards were prepared using analytical grade methanol (≥99% purity, Sigma-Aldrich, St. Louis MO, USA) in sparged natural seawater (bubbled through a sparging stone with nitrogen at 50 mL/minute, ≥5 hours at room temperature). Methanol standards were included in every run, in triplicate technical replicates, with typical concentrations in the range of 0.240–24 micromolar (μM), a typical calibration curve is represented in S1 Fig. Method detection limits were determined using the multiple injection statistical method (8 injections, 7 degrees of freedom) [16].

### Growth of cultures, cell counts, photosystem efficiency, and survey measurement of phytoplankton culture methanol

All phytoplankton culture assays were grown in biological triplicate, except where noted. In general, the methanol production surveys employed artificial seawater based media prepared with Turks Island Salt solution [17], except for *T*. *erythraeum* IMS101 cultures where RMP medium was prepared with aged, 0.2 μm filtered Sargasso Seawater as previously published [13]. Phytoplankton were inoculated from exponentially growing starter cultures, to minimize waste product carryover and growth artifacts, (1.5 mL starter culture) into 1.5L of media in acid washed Pyrex 2L reusable media bottles containing a small Teflon coated stirbar for mixing during samplings (Fisher Scientific, Pittsburgh, PA, USA). All were performed in biological triplicate. An exception was *T*. *erythraeum* IMS101 that was grown in biological duplicate in acid washed polycarbonate 2.1L Fernbach flasks prepared as previously described [13]. Phytoplankton were loosely capped to allow air exchange and assayed immediately after inoculation and throughout their growth cycle (daily near the midpoint (“algal noon”) in the light cycle), for methanol and other measurements as follows: 50 mL culture volumes were aseptically removed from each biological replicate of phytoplankton culture after being gently mixed on a stir plate at 100 RPM for 1–2 minutes, 40 mL were filtered through a 25 mm combusted Whatman GF/F glass fiber filter (GE Healthcare Bio-Sciences, Pittsburgh, PA, USA) using gentle vacuum filtration and exchange of filters as necessary to avoid artifacts from cell lysis. Filtered culture supernatants were transferred to amber pre-cleaned 40 mL EPA vials equipped with Teflon lined silicone septa (Fisher Scientific, Pittsburgh, PA, USA) and placed in the Tekmar autosampler (room temperature) for immediate analysis, with technical replicates of three for each biological replicate. In all cases process blanks of media were included of abiotic, non-inoculated controls of each type of media used and incubated alongside the phytoplankton samples. Subsamples of 1 mL were taken and stored in the dark at in situ temperatures for fast repetition rate photosystem fluorometry to determine photosynthetic efficiency (F_v_/F_m_) using a Fluorescence Induction and Relaxation (FIRe) instrument as per the manufacturer’s recommendations (Satlantic LP, Halifax, NS, Canada). Typically, cell counts were measured using a Millipore Guava flow cytometer equipped with the InCyte software package (Millipore, Billerica MA, USA) based on a previously described method [18]. However, when flow cytometry was not possible, phytoplankton samples were preserved in 1% glutaraldehyde and counted using nannoplankton chambers according to the manufacturer’s recommendations (PhycoTech Incorporated, St. Joseph, MI, USA) on a Zeiss AxioStar microscope equipped with filter cubes to monitor chlorophyll A, Acridine Orange, and 2-(4-amidinophenyl)-1H -indole-6-carboxamidine (DAPI) epifluorescence (Zeiss, Oberkochen, Germany). Axenicity of all phytoplankton cultures was tested before and after every assay using microscopy and purity medium methods as previously described [19].

### Methanol consumption assays

Isotopically enriched ^13^C-methanol (^13^CH_3_OH, 99%; Cambridge Isotope Laboratories, Tewksbury, MA, USA) was used to track the possible consumption of methanol by all microbial strains used in this study. All phytoplankton were grown in 1L batches of media identical to that used for the methanol surveys, detailed above, with the addition of ^13^C-methanol to a 2.47 μM final concentration in biological triplicate unless otherwise specified. Phytoplankton were grown until late stationary phase and sample time points were taken once per week for cell abundance, and quantification of ^13^C-methanol monitoring the diagnostic retention time and molecular ion mass (33 m/z) of the isotopically enriched substrate as detailed above. A positive control for ^13^C-methanol consumption was performed using the methyloptroph *M*. *marina* (ATCC 35842) inoculated at about 1 X 10^6^ cells per mL in 1L of f/2-Si medium amended with 2.47 μM ^13^C-methanol, in a 2L glass bottle and incubated at 23°C in a light box incubator as detailed above. Abiotic process controls were run as indicated above.

### ^13^C-bicarbonate label incorporation experiments and total carbon content analyses

In order to test if phytoplankton methanol production is derived from cellular fixed carbon *de novo*, isotopically enriched bicarbonate (^13^C-labeled sodium bicarbonate 99%, Cambridge Isotope Laboratories) incorporation experiments were performed with the phytoplankton cultures *P*. *tricornutum* and *Synechococcus* sp. 8102 with artificial seawater media identical to above, except 10% of the total sodium bicarbonate (0.214 mM final concentration of the 2.14 mM total medium bicarbonate added as a sterile addition post autoclaving) was replaced with ^13^C-bicarbonate, based on a previously established method [20]. To help prevent atmospheric carbon dioxide exchange and subsequent dilution effects of isotopically labeled bicarbonate a 0.2 μm filtered argon headspace was placed on each of the culture replicates and with each sampling event sterile argon was gently flowed into the culture flask (approximate flow rate of 10 mL per minute) and then capped tightly for incubation, as previously described [21]. Samples were assessed for purity at each time point and abiotic process controls were run as indicated above. Stable carbon isotope ratios were analyzed by the WHOI Organic Mass Spectrometry Facility using a Finnigan DeltaPlus isotope ratio mass spectrometer system (IRMS) (Thermo Fisher Scientific, Waltham, MA, USA). Total particulate organic carbon content of non-isotopically labeled cultivation experiments was determined by the WHOI Nutrient Analytical Facility using a Thermo Flash EA1112 Carbon/Nitrogen Analyzer employing Dynamic Flash Combustion (Thermo Fisher Scientific). In both cases, biological triplicate phytoplankton cultures were gently mixed on a stirplate at 100 RPM for 1–2 minutes and subsampled (volumes ranged from 5–20 mL depending on culture density) onto pre-combusted 25 millimeter GF/F filters in triplicate for each biological replicate. Cells were harvested by vacuum filtration, with care to keep the pressure differential at less than 0.5 atmosphere, the filters were then stored immediately at -20°C. Decarbonation was performed by incubating filters over fuming hydrochloric acid for 24 hours, using standard methods, and processed for final instrumental analysis.

## Results

### Phytoplankton production of methanol is polyphyletic

Phytoplankton cultures of Bacteria (cyanobacteria: *Synechococcus* sp. 8103, and *Prochlorococcus marinus*), and Eukarya (heterokont diatom:, *Phaeodactylum tricornutum*, coccolithophore: *Emiliania huxleyi*, cryptophyte: *Rhodomonas salina*, and non-diatom heterokont: *Nannochloropsis oculata*) lineages were chosen based on their ecological representation in the coastal or open ocean environments, amenability to laboratory cultivation and, most importantly, their axenicity. All phytoplankton cultures displayed near- or multi-micromolar amounts of methanol upon reaching stationary phase, Table 1, well above background abiotic controls that were in the 20–50 nanomolar range typical for ambient background atmospheric methanol present in media blanks or initially inoculated and measured cultures. Cell counts and morphologies of our stationary phase phytoplankton cultures were all within the expected ranges of what previously published observations have shown [13, 14, 22] and all cultivars were determined to be homogeneous and free of contaminating microbial associates, excluding *T*. *erythraeum* IMS101 which was a mixed culture to start with. The amount of methanol normalized to cell abundance varied up to 100-fold among cultivars tested and the cyanobacteria *Synechococcus* and *Prochlorococcus* displayed the lowest amount of methanol per cell, Table 1. Carbon content per cell was measured for *R*. *salina*, *N*. *oculata*, and *P*. *tricornutum* at the time of maximal methanol production and varied from 2–3 fold among isolates tested (Table 1).

**Table 1:** Seven phytoplankton cultures screened for methanol production at stationary phase normalized to cell abundances.

Phytoplankton culture	MeOH Prod (days)	Average uM MeOH	Cells/mL	fmol MeOH/cell	pmol C/cell	%MeOH/total cellular carbon
***Rhodomonas salina* CCMP1319**	15	1.4 (+/-) 0.2	1.9 X 10^5^ (+/-) 2.4 X 10^4^	0.7 (+/-) 0.2	2.8 (+/-) 0.4	0.3 (+/-) 0.1
***Nannochloropsis oculata* CCMP525**	18	2.9 (+/-) 0.1	7.4 X 10^6^ (+/-) 0.9 X 10^6^	0.39 (+/-) 0.05	0.26 (+/-) 0.14	0.09 (+/-) 0.03
***Phaeodactylum tricornutum* CCMP632**	24	12.9 (+/-) 3.4	4.5 X 10^6^ (+/-) 1.9 X 10^5^	2.9 (+/-) 0.3	1.4 (+/-) 0.5	0.2 (+/-) 0.1
***Emiliania huxleyi* CCMP371**	14	13.7 (+/-) 2.0	2.6 X 10^6^ (+/-) 2.0 X 10^5^	5.3 (+/-) 1.0	ND	ND
***Trichodesmium erythraeum* IMS101**	14	5.5 (+/-) 0.2	1.0 X 10^6^ (+/-) 2.0 X 10^5^	5.0 (+/-) 2.0	ND	ND
***Synnechococcus* sp. WH8103**	10	0.8 (+/-) 0.1	1.4 X 10^7^ (+/-) 1.8 X 10^6^	0.06 (+/-) 0.01	ND	ND
***Prochlorococcus marinus* MED4ax**	10	1.1 (+/-) 0.1	2.2 X 10^7^ (+/-) 2.0 X 10^6^	0.05 (+/-) 0.01	ND	ND

Values for cultivars *R*. *salina*, *N*. *oculata*, and *P*. *tricornutum* represent the observed maxima for methanol production from time-course samplings and were normalized to total cellular carbon at that particular time point. All cultivars were axenic, except for *T*. *erythraeum* IMS101 which was only available as a xenic culture.

### Methanol is produced in a punctuated fashion

**Fig 1:**
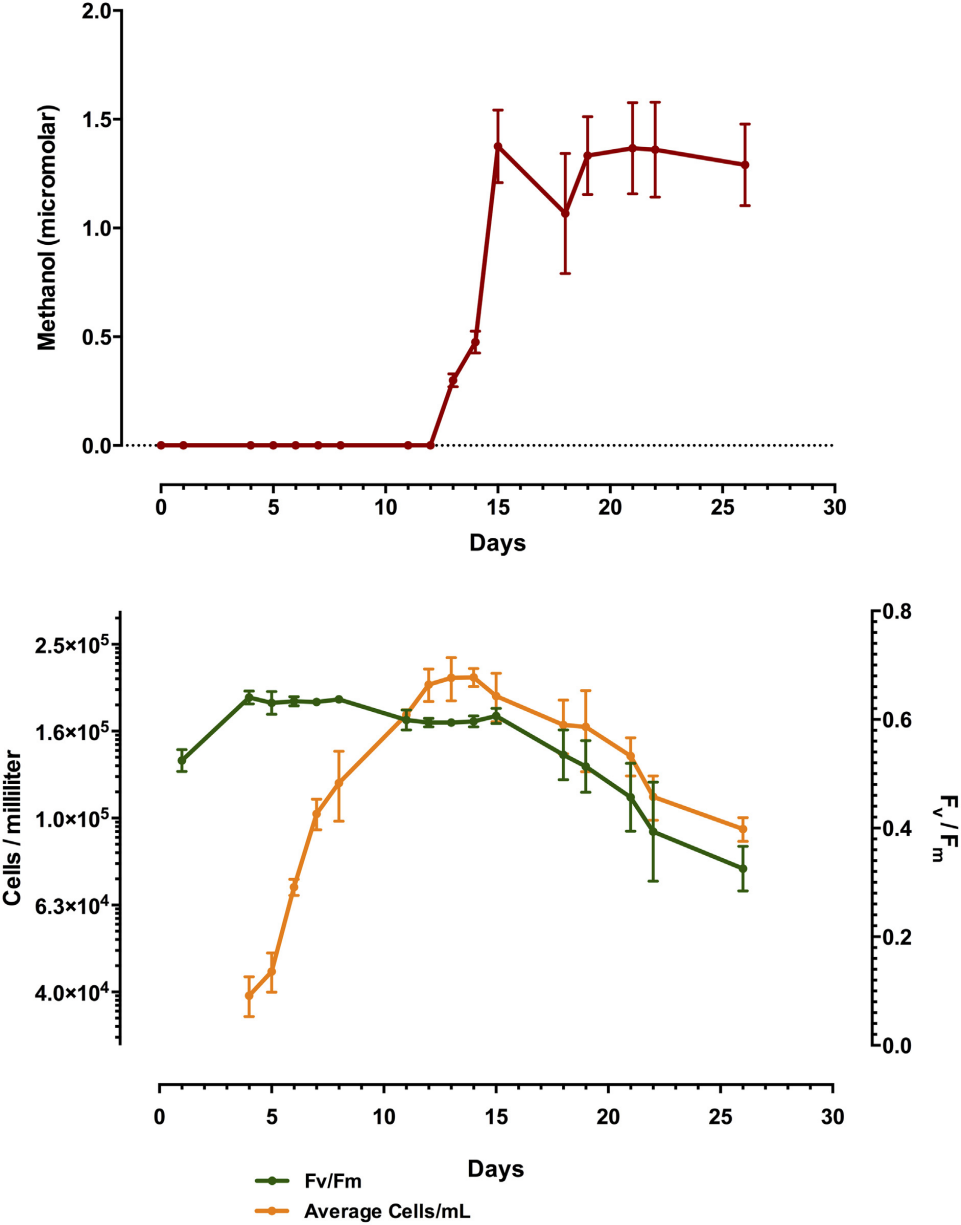
Time-course plot of *R*. *salina* batch culture grown in biological triplicate showing methanol production (y-axis) over time (x-axis) in micromolar amounts in upper panel, and cell abundances and corresponding photosystem health (F_v_/F_m_) in lower panel. Note, clear methanol production coinciding with the onset of stationary phase.

**Fig 2:**
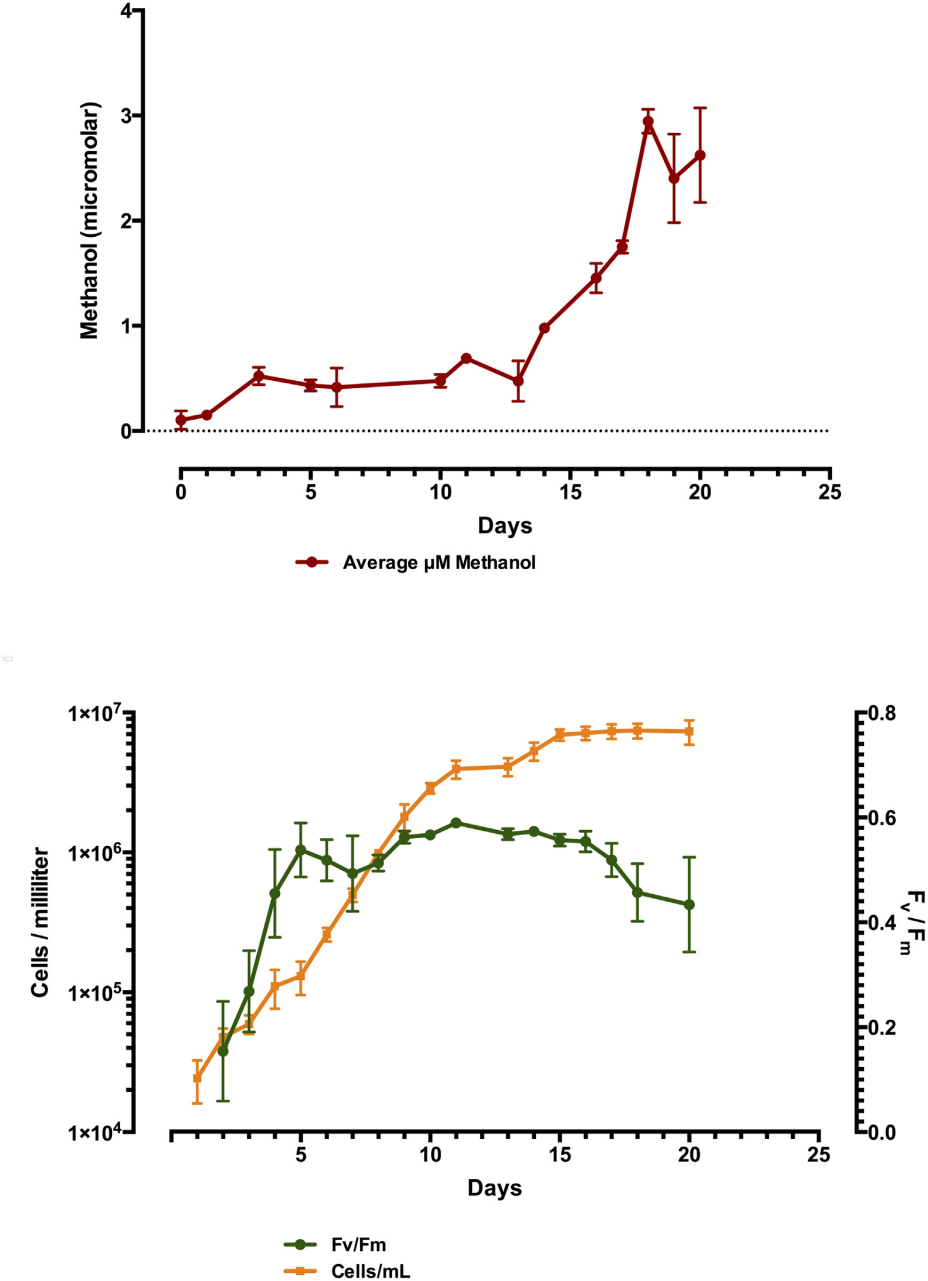
Time-course plot of *N*. *oculata* batch culture grown in biological triplicate showing methanol production (y-axis) over time (x-axis) in micromolar amounts in upper panel, and cell abundances and corresponding F_v_/F_m_ in lower panel. Note, highest methanol production where cell abundance levels off.

**Fig 3:**
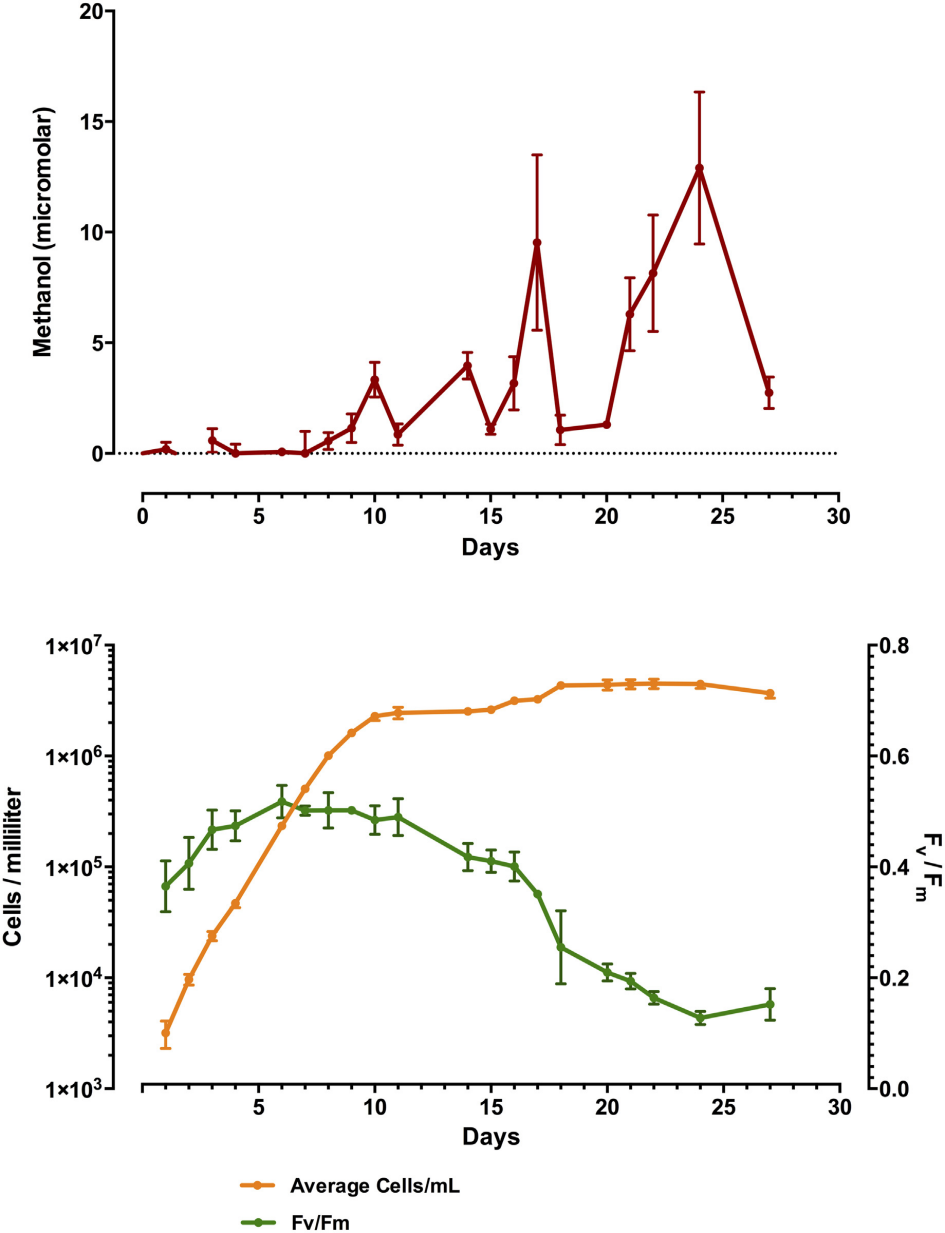
Time-course plot of *P*. *tricornutum* batch culture grown in biological triplicate showing methanol production (y-axis) over time (x-axis) in micromolar amounts in upper panel, and cell abundances and corresponding F_v_/F_m_ in lower panel. Note, spikes of methanol production and apparent loss coincidental with maximal cell abundance and drop in F_v_/F_m_.

Phytoplankton methanol production was tracked with time in triplicate batch cultures of *R*. *salina*, *N*. *oculata* and *P*. *tricornutum*, Figs 1, 2 and 3, respectively. Time-course measurements of methanol production in all cultures tested displayed the general trend of increased methanol production over time, in the case of *R*. *salina* production was quite punctuated, correlating with the very end of the exponential growth phase, where cell counts plateaued at about 2 X 10^5^ cells/mL (Fig 1). A similar trend was observed with *N*. *oculata*, where the presence of methanol in the culture medium more than doubled from day 13 to day 16 up to its maximum of 2.9 μM at day 18 with only a slight increase in cellular abundance over that time period of 1.3 X 10^6^–1.8 X 10^6^ (Fig 2). Methanol measured in the culture medium of *P*. *tricornutum* showed a significant increase correlating with early stationary phase at day 10, however, the maximum amount of culture medium methanol was observed two weeks later on day 24, with three significant spikes in methanol production and apparent loss from the culture medium (Fig 3). In general, F_v_/F_m_ measurements determined by FIRe, decreased with increasing amounts of methanol present in the culture medium. Additionally, the replicate cultures of *P*. *tricornutum* displayed large variations in methanol measured in the filtered medium, with significant losses of methanol occurring between days 17–18 and days 24–27 with variations in methanol on the order of 5 μM. Further experiments were conducted, see below, in an attempt to explain this perplexing result. We suspected the methanol loss was due to a methylotrophic bacterial contaminant in the *P*. *tricornutum* replicate cultures, however, these algal cultures proved axenic in every case (data not presented). Moreover, in every culture tested for methanol production in this initial survey, all phytoplankton remained axenic throughout the time course, with the exception of *T*. *erythraeum* which was only available in xenic form.

### Methanol is a waste product of phytoplankton

Assays monitoring ^13^C-labeled methanol loss in phytoplankton cultures were compared to an abiotic control over an entire growth cycle period of 14–24 days, depending upon cultivar, under conditions otherwise identical to our methanol production growth conditions (S1 Table). In all cases, no ^13^C-methanol loss was detectable within our 10% measurement errors versus comparable abiotic controls (all samples were generally within 20–50 nM of one another), with the exception of the xenic *T*. *erythraeum* IMS101, suggesting that phytoplankton cultivars tested were not capable of any appreciable methanol consumption during their growth cycle and that methanol is indeed a true waste product of phytoplankton, similar to higher plants [7]. In stark contrast, our positive control (the methylotroph *M*. *marina*) displayed no detectable methanol in our earliest time point after three days (S1 Table). Cultivar *T*. *erythraeum* IMS101 displayed modest methanol consumption of ^13^C-methanol (~500 nM) after 14 days growth, presumably due to the epibiotic microbial consortium and not from the cyanobacterium. Interestingly, no ^13^C-labeled methanol consumption was observed in the case of *P*. *tricornutum* where significant ^12^C-methanol loss from the culture medium was observed at specific periods in the sampling time-course, Fig 3. To further characterize this loss, we spiked cultures of *P*. *tricornutum* with ^13^C-methanol and measured it simultaneously in Scan/Single Ion Monitoring (SIM) mode, monitoring the m/z = 31 base peak ion (diagnostic for the stable H2C = OH+ ion) and m/z = 33 ions (diagnostic for the ^13^C-labeled molecular ion, M+, of methanol). Interestingly, the m/z = 31 methanol measurements were again significantly variable over time and the m/z = 33 ion remained constant relative to the abiotic control (S2A and S2B Fig). Moreover, we noticed that the variability in the ^12^C-methanol measurements coincided with the sinking of the cells and the presence of micro bubbles in the culture of *P*. *tricornutum* that released with gentle mixing from the newly formed diatom mat at the bottom of the culture bottle and present in larger form in the “mucoid slick” layer at the top of the culture.

### Methanol is derived from algal biomass

In order to test if methanol production was derived from algal biomass fixed during culture growth, we added ^13^C- labeled bicarbonate at 10% of the total bicarbonate added to our artificial seawater composition and monitored the labeled methanol production. In parallel, ^13^C-bicarbonate incorporation was measured in the particulate cellular fraction of cultures *P*. *tricornutum* and *Synechococcus* sp. 8102 (note strain difference from Table 1), using isotope ratio mass spectrometry (IRMS). We observed cellular incorporation of labeled bicarbonate to be consistent at about 10% efficiency with the overall yields of labeled methanol to be about 10% of what had been observed (Table 2), consistent with the idea that methanol is indeed produced from phytoplankton biomass labeled *de novo*.

**Table 2:** Isotope ratio measurements of axenic phytoplankton cultivars grown in defined medium containing 10% of the total bicarbonate as the isotopically labeled form (^13^C).

Phytoplankton Culture	Growth time (days)	Average μM Methanol	Average Cell counts /mL	fmol ^13^C-MeOH/cell	pmol ^13^C-Carbon/cell	%^13^C-MeOH/^13^C-Carbon/cell
***Synechococcus* sp. WH8102**	0	ND	~1 x 10^3^	ND	ND	ND
	18	0.29 (+/-) 0.13	1.2 x 10^7^ (+/-) 2.0 x 10^6^	0.024 (+/-) 0.01	0.0078 (+/-) 0.003	0.3 (+\-) 0.1
***Phaeodactylum tricornutum* CCMP632**	0	ND	~1 x 10^3^	ND	ND	ND
	23	0.49 (+/-) 0.10	3.9 x 10^6^ (+/-) 2.2 x 10^5^	0.13 (+/-) 0.05	0.06 (+/-) 0.03	0.2 (+/-) 0.1

Phytoplankton were grown in biological triplicate with technical triplicate measurements performed on each culture (n = 9).

Abiotic negative controls of ^13^C-labeled bicarbonate added at 10% of the total bicarbonate in artificial seawater medium yielded no detectable ^13^C-labeled methanol, and no particulate organic carbon was detected above background. Fractional abundances (^13^C/(^12^C + ^13^C)) were calculated for *P*. *tricornutum* and *Synechococcus* sp. 8102 particulate organic carbon samples. Both cultures displayed full incorporation of the 10% ^13^C-bicarbonate with fractional abundances of 0.113 (+/-) 0.010 (23 days growth) for *P*. *tricornutum* and 0.103 (+/-) 0.007 (18 days growth) *Synechococcus* sp. 8102. Isotopically heavy methanol was measured for these same time points in these biological triplicate cultures: *P*. *tricornutum* displayed 0.49 (+/-) 0.10 μM and *Synechococcus* sp. 8102 displayed 0.29 (+/-) 0.13 μM methanol, quantified using a ^13^C-labeled methanol standard curve dilution series of 24.67, 2.47, and 0.25 μM. Overall, visible and uniform growth and cell counts were comparable among Tables 1 and 2 and overall ratios of ^13^C-methanol/^13^C-cellular carbon were 0.3 (+/-) 0.1% and 0.2 (+/-) 0.1% respectively for *Synechococcus* sp. 8102 and *P*. *tricornutum* (this diatom culture showed very good agreement with the ^12^C-methanol/^12^C-cellular carbon measurements in Table 1). Negative controls performed with *Synechococcus* sp. 8102 and *P*. *tricornutum* grown in the absence of ^13^C-bicarbonate label displayed no detectable peaks with similar retention time (at or near 13.1 minutes (+/-) 2 minutes), ion signal of 33 m/z, or similar fragmentation pattern to 13C-methanol were detected (data not shown).

### Possible enzymatic methanol production

**Fig 4:**
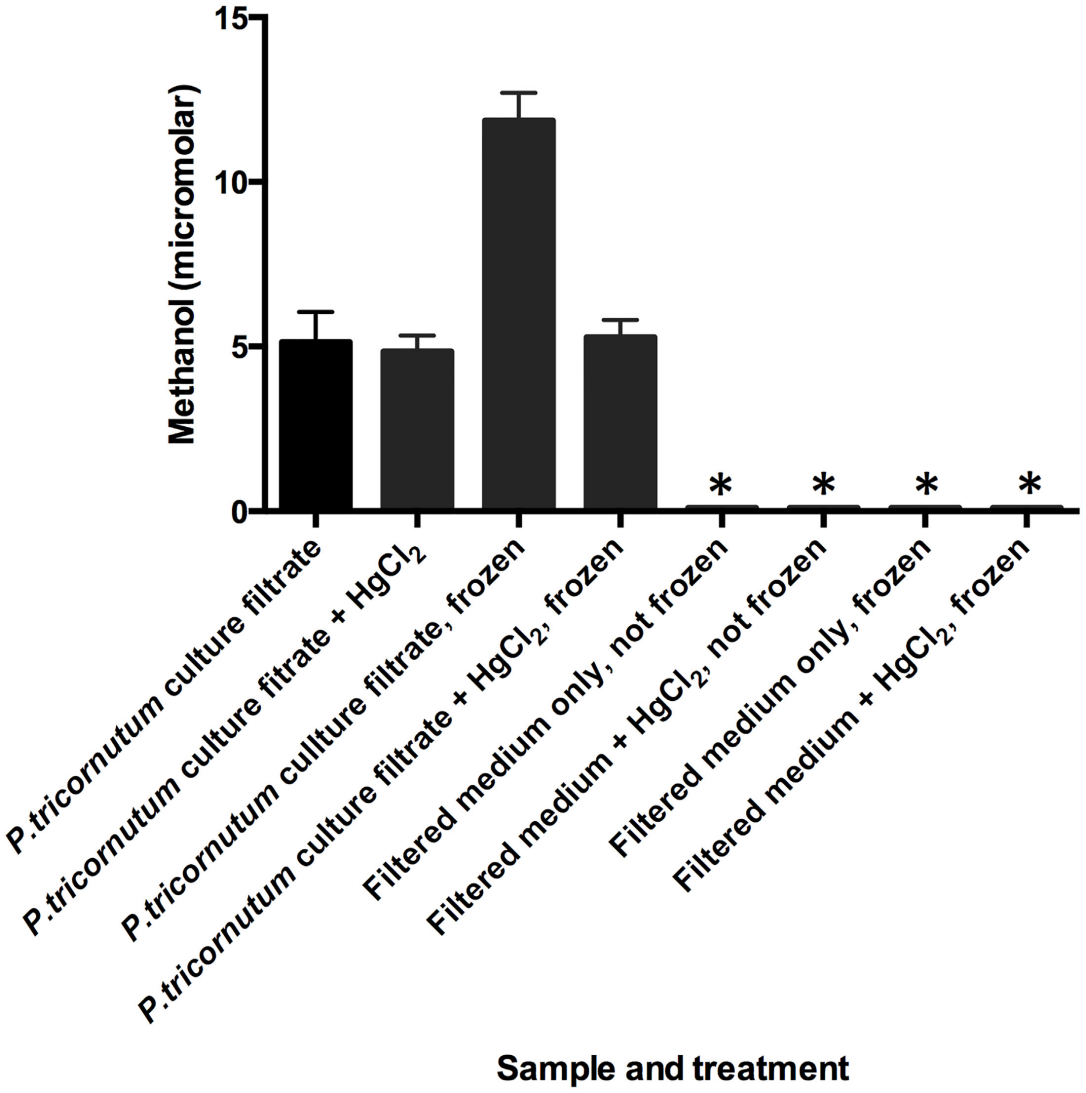
*Phaeodactylum tricornutum* CCMP632 culture sample after 10 days growth (conditions detailed in Methods, with biological duplicates and technical triplicate measurements) were tested for background production of methanol after filtering through GF/F filters and freezing at -20°C for 24 hours. Samples were processed with or without 4mM mercuric chloride (final concentration, as indicated), incubated for 10 minutes at room temperature then processed as detailed in Methods. A subset of samples was frozen at -20°C for 24 hours, another was measured immediately. Blanks consisting of negative media controls were processed in parallel. Asterisks indicate detection of background methanol at approximately 100 micromolar or less.

An important precaution that we took was to ensure all samples were measured immediately after filtering and not cryopreserved. In rare cases we noticed that frozen samples (-20°C) compared to freshly measured replicates possessed up to approximately 2 X higher amounts of methanol (this was particularly evident in axenic cultures of *P*. *tricornutum*). Upon further analysis, this artificial background was eliminated with the addition of 4mM final concentration mercuric chloride as a preservative (Fig 4) which completely suppressed the background methanol production in this case. In general, background methanol production was prevented by measuring immediately and staggering technical replicates in the P&T-GC/MS autosampler assured that background methanol production did not occur over the time course of each analysis. Additionally, this background production was only observed post cryopreservation, when observed at all, and not when samples were stored overnight at room temperature, even for *P*. *tricornutum* samples.

## Discussion

We report here the first quantitative measurements of methanol production from a broad phylogenetic array of phytoplankton. Special care was taken to insure that the phytoplankton cultures analyzed were axenic (the exception being *T*. *erythraeum* IMS101 which is notoriously difficult to maintain in pure culture) and that methanol production was measured in only viable, physiologically intact cell cultures with functional photosystems, as opposed to decaying necromass containing heterotrophic microbes. In all cases, no loss of ^13^C-labeled methanol was detected, suggesting that methanol is a true phytoplankton waste product (*T*. *erythraeum* IMS101 is again the exception, displaying modest consumption, presumably due to methylotrophic microbial associates in the culture).

The notion that methanol could be produced by phytoplankton first came to our attention through the initial discovery of an abundant clone sequence (~20% of the total sequenced clones) of a putative methylotroph (100% identity to *Methylophaga marina* compared over >600bp) associated with a culture of *Trichodesmium thiebautii* H94, (maintained in continuous culture since 2004) during a small-subunit ribosomal RNA gene (rDNA) sequence survey [23, 24]. Axenicity of phytoplankton cultures was a priority in our study because the presence of heterotrophic microbial associates in phytoplankton cultures presented the null hypothesis that phytoplankton might not be the actual methanol producers, rather, this OVOC could be liberated by catabolic community members as had been posited previously [25]. However, our data suggest that the inverse can also occur, where phytoplankton epibionts can possess the capacity to consume methanol and thus, as in flowering plants [7], methanol could have profound implications on phytoplankton interactions with methylotrophic microbial communities.

Initially regarded as a curiosity, methylotrophy has proven to be a major metabolic process on the planet as many more methylotrophs are discovered [2, 26]. Particularly intriguing is the abundance of specialists such as the OM43 clade of beta-proteobacteria dependent upon methanol as a sole carbon and energy source in the marine environment [6]. Interestingly, the OM43 clade of obligate methylotrophs have been found to dominate in diatom blooms [27] and one of the most highly detected proteins from coastal upwelling marine and Antarctic waters was methanol dehydrogenase with highest similarity to the abundant OM43 methylotroph clade of bacteria [28, 29]. Indeed, macro red and brown algal samples have served as a common inoculum for isolating methylotrophic bacteria from the marine environment [30]. Methylotroph-phytoplankton interactions could prove to be as intricate as microbial interactions with specific terrestrial plants where methylotrophs such as *Methylobacter* spp. colonize the underside of leaves and have been shown to produce plant growth hormones which are involved in the health of the overall holobiont [31, 32].

It has been suggested that a succession of microbes could be involved in methanol production in the water column with the interaction of dissolved organic matter (DOM) [25]. This downstream processing of primary production could be an important source of methanol, particularly in the aphotic zone. Our data provide strong evidence for a pathway leading from bicarbonate to methanol that could be a dominant source of this OVOC in the photic zone. Furthermore, our preliminary observations suggest that the possible enzymatic activity from the diatom *P*. *tricornutum* is quite refractory, being activated by a freeze-thaw cycle, as we observed, Fig 4. If this is a widespread trait among phytoplankton, methanol production could persist in sinking phytoplankton detritus, thus influencing deeper regions of the ocean in areas where detrital sinking rates are high, such as productive coastal upwelling regions.

Our data suggest that phytoplankton have the potential to provide significant amounts of methanol in the world’s oceans. Methanol production was as high as 0.3% of the total cellular carbon produced in a culture. We made efforts to estimate methanol production in terms of primary productivity as has been previously measured for plants [33]; however, this proved difficult due to the pulsed production of methanol in our culture systems. We recommend future attempts at these types of measurements employing chemostat growth conditions to pinpoint high and low methanol production growth conditions.

Considering *Prochlorococcus* (one of the phytoplankton cultures displaying the lowest amounts of methanol in our study), this genus alone could contribute approximately 846–1693 Tg of methanol per year based upon our measurements (assuming from our data, an average turnover time of 1–2 days and methanol production of 0.05 femtomoles (1.6 femtograms) methanol per cell combined with the yearly mean of 2.9 X 10^27^ *Prochlorococcus* global population size, as estimated previously [34]. This amount of methanol production is on the order of alkane production recently estimated for *Prochlorococcus*, which showed a range of 0.466–0.711 femtograms total alkane production per cell, corresponding to 269–539 Tg annually (35). Terrestrial plants have been estimated to produce about 75 Tg of methanol per year on average (36), although this is a poorly constrained value with a range from 50–350 Tg, primarily due to an incomplete understanding of source and sink strengths [3]. Nevertheless, with *Prochlorococcus* and *Synechococcus* accounting for 25% of gross primary productivity in the ocean as previously estimated [34], our estimates of phytoplankton methanol production could rival or exceed terrestrial sources. Complicating factors such as the punctuated nature of methanol production towards the end of the cell cycle, longer turnover rates for other phytoplankton lineages, and unknown factors such as how methanol production varies with specific strains and nutrient limitation could lower these estimates significantly.

Losses of methanol due to the culture system in our study not being completely closed are likely negligible, as estimated from our ^13^C-methanol abiotic controls, except for the case of *P*. *tricornutum*, discussed below. However, we have noted significant amounts of methanol liberated from unpreserved frozen cultures (Fig 4), suggesting that decaying phytoplankton detritus could contribute significantly to the methanol budget deeper in the water column. Although beyond the scope of the present study, future work is planned to measure methanol from sinking detritus which could be an important source of C1 compounds fueling methylotrophy in the deep oceans, analogous to the evolution of methanol from decaying plant material on land [35].

We were surprised to observe a significant loss of methanol from our *P*. *tricornutum* phytoplankton culture system. One of the main reasons for choosing the P&T-GC/MS instrumentation for measuring methanol was its superior detection ability for this very polar OVOC which is highly soluble in water and behaves as an ideal mixture when at a low molar fraction (*i*.*e*. methanol: water <<0.1) [36]. In fact, P&T-GC/MS has been successfully used by our group as well as other workers for measurement of methanol and other polar VOCs in gas well production fluid brines and saline pore-water samples [15, 37]. Significant methanol loss was observed during the evolution of visible capillary bubbles in cultures of *P*. *tricornutum*, coinciding with spikes in methanol production which also corresponded with the cells sinking to the bottom of the culture flask. Technical replication error of our *P*.*tricornutum* measurements was negligible (within 5% or less of each other), leaving the biological replicates responsible for all the significant error in this case. We speculate that the highest production of methanol is during this phase of culture morphological change and is a byproduct of some sort of cell biopolymer tailoring or maturation, similar to the changing of the pectin macromolecule in higher plants [7]. Although beyond the scope of this study and the subject of further investigation, we further speculate that the stirring and filtration method over the GF/F glass fiber filter solubilized methanol into the medium at early phases of bubble formation, however, once the bubbles coalesced and rose to the top of the batch culture they were then free to escape and export from the system, particularly during oxygen saturation growth conditions. This opens up a previously unexpected possibility of methanol export to the atmosphere as gas bubbles would rise relatively quickly and have little chance for methylotrophic microbial consumption.

The mechanism of methanol production in phytoplankton warrants further investigation. Additions of 4mM mercuric chloride, previously shown to be an effective preservative and inhibit enzymatic reactions [38], obliterated extracellular production of methanol. Methanol production in phytoplankton is sure to differ mechanistically from terrestrial “higher” plants due to the lack of pectin in the cultures used in this study and in most other phytoplankton (with the possible exception of some members of the green algae) [39]. In phytoplankton, methoxy, sulfoxy, and ethoxy groups are known to add gel stability to polysaccharides by aiding the formation of a double helical chain geometry [40]. Although the polysaccharides of algae are very diverse in monomeric subunits and linkages, the conserved theme of methoxylation predominates [41]. Other substrates for enzymatic methanol production exist such as dimethylsulfoniopropionate (DMSP) demethylation, or hydrolysis of glycine betaine to trimethylamine and subsequent demethylation to di- and monomethylamine [3] but these compounds are not consistently produced among the broad array of phytoplankton that we surveyed, and if so only in small quantities. Thus, demethoxylation of abundant cellular polysaccharides is an explanation that is most consistent with any observation of methanol production in relatively high amounts across a polyphyletic array of axenic phytoplankton.

The timing of methanol production with early stationary phase, inflection of F_v_/F_m_ and presumably nutrient depletion in the cultures we measured (*R*. *salina*, *N*. *oculata*, and *P*. *tricornutum*) warrants further investigation. If this type of pattern also occurs during phytoplankton blooms then this could provide extraordinary pulses of methanol for heterotrophic consumption and possibly as an atmospheric source.

## Conclusions

In this study, we report methanol production in the micromolar range from a polyphyletic collection of axenic phytoplankton cultures. Furthermore, methanol does not appear to be consumed by these axenic strains and is most likely a waste product of the phytoplankton growth process, with punctuated methanol production at a maximum during early stationary phase, observed in cultivars that we measured over a time course. Stable isotope labeled bicarbonate incorporation experiments suggest that methanol is produced *de novo* from algal biomass and comparative elemental analyses showed that methanol accounts for a significant portion of phytoplankton cellular carbon. Altogether, our data suggest that phytoplankton are a major source of methanol in the marine euphotic zone. Future work remains to elucidate the mechanism of production and constrain the overall budget of marine sources and sinks of this important OVOC in the marine environment.

## Supporting Information

S1 Fig Methanol standard calibration curve. (XLSX)

S2 Fig ^12^C- and ^13^C-methanol measurements of *Phaeodactylum tricornutum*. (XLSX)

S1 File Complete parameters of P&T concentration instrumentation. (PDF)

S1 Table ^13^C-methanol consumption data for all phytoplankton studied. (XLSX)
