# Supplementary material for: Methanol Production by a Broad Phylogenetic Array of Marine Phytoplankton
Source: PLoS One. 2016 Mar 10;11(3):e0150820. doi: 10.1371/journal.pone.0150820 (PMC4786210; doi:10.1371/journal.pone.0150820)
Supplement: S1 File — (PDF) [file pone.0150820.s003.pdf]

C:\Program Files\Teledyne Tekmar\VOC TekLink\3.2\Methods\VOAwater_MOD-TM-2.msvw

SOLATek 72 (with Stratum Concentrator) Water Method

Variable	Value
Rinse Water Temp.	90°C
Sample Cup Temp.	30°C
Sample Needle Temp.	30°C
Transfer Line Temp.	125°C
Soil Valve Temp.	125°C
Sample Sweep Time	0.50 min.
Needle Rinse Volume	5 mL
Needle Sweep Time	0.50 min.
Bake Rinse Volume	7 mL
Bake Sweep Time	0.50 min.
Bake Drain Time	0.50 min.
Number of Bake Rinses	1
Valve Oven Temp.	150°C
Transfer Line Temp.	150°C
Sample Mount Temp.	90°C
Purge Ready Temp.	45°C
Condenser Ready Temp.	40°C
Condenser Purge Temp.	20°C
Standby Flow	0 mL/min.
Pre-Purge Time	0.50 min.
Pre-Purge Flow	40 mL/min.
Sample Heater	Off

Variable	Value
Sample Preheat Time	1.00 min.
Sample Temp.	40°C
Purge Time	11.00 min.
Purge Temp.	0°C
Purge Flow	40 mL/min.
Dry Purge Time	1.00 min.
Dry Purge Temp.	40°C
Dry Purge Flow	200 mL/min.
GC Start	Start of Desorb
Desorb Preheat Temp.	245°C
Desorb Drain	On
Desorb Time	2.00 min.
Desorb Temp.	250°C
Desorb Flow	300 mL/min.
Bake Time	2.00 min.
Bake Temp.	260°C
Bake Flow	400 mL/min.
Condenser Bake Temp.	175°C
Focus Temp.	-190°C
Inject Time	1.00 min.
Inject Temp.	175°C
Standby Temp.	35°C

Notes

(none)
